# Automated quantification of myocardial tissue characteristics from native T_1_ mapping using neural networks with uncertainty-based quality-control

**DOI:** 10.1186/s12968-020-00650-y

**Published:** 2020-08-20

**Authors:** Esther Puyol-Antón, Bram Ruijsink, Christian F. Baumgartner, Pier-Giorgio Masci, Matthew Sinclair, Ender Konukoglu, Reza Razavi, Andrew P. King

**Affiliations:** 1 School of Biomedical Engineering & Imaging Sciences, King’s College London, Rayne Institute, 4th Floor Lambeth Wing St Thomas Hospital, Westminster Bridge Road, London, SE1 7EH UK; 2 Department of Adult and Paediatric Cardiology, Guy’s and St Thomas’ NHS Foundation Trust, London, UK; 3 Computer Vision Lab, ETH Zürich, Sternwartstrasse 7, Zürich, Switzerland; 4 Biomedical Image Analysis Group, Department of Computing, Imperial College London, 3rd floor Huxley Building, 180 Queen’s Gate, London, SW7 2AZ UK

**Keywords:** Native T_1_ mapping, Convolutional neural networks, Automatic analysis, Cardiac MR Segmentation, Quality control, UK Biobank

## Abstract

**Background:**

Tissue characterisation with cardiovascular magnetic resonance (CMR) parametric mapping has the potential to detect and quantify both focal and diffuse alterations in myocardial structure not assessable by late gadolinium enhancement. Native T_1_ mapping in particular has shown promise as a useful biomarker to support diagnostic, therapeutic and prognostic decision-making in ischaemic and non-ischaemic cardiomyopathies.

**Methods:**

Convolutional neural networks (CNNs) with Bayesian inference are a category of artificial neural networks which model the uncertainty of the network output. This study presents an automated framework for tissue characterisation from native shortened modified Look-Locker inversion recovery ShMOLLI T_1_ mapping at 1.5 T using a Probabilistic Hierarchical Segmentation (PHiSeg) network (PHCUMIS 119–127, 2019). In addition, we use the uncertainty information provided by the PHiSeg network in a novel automated quality control (QC) step to identify uncertain T_1_ values. The PHiSeg network and QC were validated against manual analysis on a cohort of the UK Biobank containing healthy subjects and chronic cardiomyopathy patients (N=100 for the PHiSeg network and N=700 for the QC). We used the proposed method to obtain reference T_1_ ranges for the left ventricular (LV) myocardium in healthy subjects as well as common clinical cardiac conditions.

**Results:**

T_1_ values computed from automatic and manual segmentations were highly correlated (r=0.97). Bland-Altman analysis showed good agreement between the automated and manual measurements. The average Dice metric was 0.84 for the LV myocardium. The sensitivity of detection of erroneous outputs was 91%. Finally, T_1_ values were automatically derived from 11,882 CMR exams from the UK Biobank. For the healthy cohort, the mean (SD) corrected T_1_ values were 926.61 (45.26), 934.39 (43.25) and 927.56 (50.36) for global, interventricular septum and free-wall respectively.

**Conclusions:**

The proposed pipeline allows for automatic analysis of myocardial native T_1_ mapping and includes a QC process to detect potentially erroneous results. T_1_ reference values were presented for healthy subjects and common clinical cardiac conditions from the largest cohort to date using T_1_-mapping images.

## Background/Introduction

Cardiovascular magnetic resonance (CMR) provides insights into myocardial structure and function noninvasively, with high diagnostic accuracy and without ionising radiation. Late Gadolinium Enhancement (LGE) has become the reference standard for non-invasive imaging of myocardial scar and focal fibrosis in both ischaemic [2] and non-ischaemic cardiomyopathy [3]. LGE is useful in cardiac conditions which have stark regional differences within the myocardium, but it cannot correctly visualise myocardial pathology that is diffuse in nature and affects the myocardium uniformly. Examples include diffuse myocardial inflammation, fibrosis, hypertrophy, and infiltration [4]. In contrast, native T_1_-mapping provides quantitative myocardial tissue characterisation, without the need for gadolinium [5]. Previous work has shown that T_1_ mapping can help to detect diffuse myocardial disease in early disease stages and aids in diagnosing the diseases’ underlying cardiac dysfunction.

Despite its recognised potential, T_1_ mapping analysis typically requires time-consuming manual segmentation of T_1_ maps. Moreover, external factors, such as hematocrit and blood flow, impact the obtained values and create variability that reduces the ability to separate healthy from diseased myocardium. Several blood correction models have been proposed to limit the impact of external factors [6–8]. However, these methods have not been evaluated in large cohort studies. Automating T_1_ analysis of myocardial tissue characterisation sequences could facilitate the clinical use of T_1_ mapping and unlock the potential to obtain T_1_ data in large populations.

In recent years, deep learning methods have shown great success in segmenting anatomical and pathological structures in medical images [9–11]. For many tasks, their accuracy is comparable to human-level performance, or even surpasses it. In the context of CMR imaging, semi-automatic and automatic techniques for cardiac cine [9, 10] and flow [12] imaging have been developed. One paper has proposed an automated segmentation method for native T_1_ maps [11]. However, this method only extracted global left ventricle (LV) myocardial T_1_ values, whereas regional assessment of septal and/or focal lesion T_1_ values is typically used to characterise diseases [13, 14]. Furthermore, T_1_ values were only reported for healthy subjects and a pooled group of cardiovascular diseases (CVD), without distinguishing between different myocardial disease processes and these values were not corrected for myocardial blood volume. In this paper we add further insight into the aforementioned areas.

Medical segmentation problems are often characterised by ambiguities, some of them inherent to the data such as poor contrast, inhomogeneous appearance and variations in imaging protocol, and some due to inter- and intra-observer variability in the annotated data used for training. To limit the effect of these factors and detect failed cases, some groups have proposed to incorporate quality control (QC) techniques [10, 11, 15]. We believe that modeling uncertainty at a per-pixel level is an important step in understanding the reliability of the segmentations and increasing clinicians’ trust in the model’s outputs. Several works have investigated uncertainty estimation for deep neural networks [16–18]. A popular approach to account for the uncertainty in the learned model parameters is to use variational Bayesian methods, which are a family of techniques for approximating Bayesian inference over the network weights. Budd *et al* [19] proposed to use this approach to automatically estimate fetal Head Circumference from Ultrasound imaging and provide real-time feedback on measurement robustness. Another approach is to probabilistic graphical models to model the conditional segmentation masks given an input image using a conditional variational autoencoder (cVAE) approach. Recently, Kohn [20] proposed a framework that combines the cVAE framework with a U-Net architecture to generate an unlimited number of realistic segmentation samples. These methods can be used to automatically segment the anatomy of interest, but additionally provide a pixel-wise uncertainty map of the confidence of the model in segmenting the input image.

In this paper, we develop a tool for automated segmentation and analysis of T_1_ maps. We use the Probabilistic Hierarchical Segmentation (PHiSeg) network [1] to segment the images, and additionally use the generated uncertainty information in a novel QC process to identify uncertain (and potentially inaccurate) segmentations. To the best of our knowledge this is the first time that segmentation uncertainty information has been used for QC in medical image segmentation. By incorporating this QC process, our framework automatically controls the quality of the segmentations and rejects those that are uncertain. We hypothesise that this method can be used to derive high quality T_1_ data without human interaction from large-scale databases. Using the proposed method we compute mean global and regional native T_1_ values from 11,882 subjects from the UK Biobank, which represents the largest cohort for T_1_ mapping images to date. We report reference values for healthy subjects and interrogate typical values obtained in important relevant subgroups of cardiomyopathies. In addition, we investigate if a blood correction model for T_1_ [6] provides better discrimination between healthy and diseased myocardium. Therefore, the contributions of this paper are threefold: novel uncertainty-based QC in the context of T_1_ analysis; investigation of myocardial T_1_ blood correction in a large-scale database; and the reporting of disease-specific reference values for the T_1_ ShMOLLI sequence.

## Materials and methods

### UK biobank dataset

CMR imaging was carried out on a 1.5 T scanner (Siemens Healthineers, Erlangen, Germany). For each subject, the ShMOLLI (WIP780B) was used to perform native (non-contrast) myocardial T_1_ mapping in a single mid-ventricular short axis (SAx) slice (TE/TR/flip-angle (FA): 1.04ms/ 2.6ms/ 35^∘^, voxel size 0.9 x 0.9 x 8.0 mm). The matrix size of all images was unified to 192 x 192. T_1_ parametric maps, with pixel-by-pixel computation of the T_1_ values, were generated using the inline reconstruction software installed on the scanner. Details of the full image acquisition protocol can be found in [21].

From the UK Biobank database, we first select a cohort of healthy subjects excluding any subjects with a history of CVD, cardiovascular risk factors, other systemic diseases or those taking medication for any systemic disease (see all exclusion criteria in Additional file 1: Table 1). Healthy subjects were identified as anyone with a body mass index (BMI) <30 kg/m^2^ and obese subjects were identified as anyone with a BMI ≥30 kg/m^2^. From the healthy group we excluded anyone with a 10-year Framingham Risk Score [22] above 10% to restrict the group to only the healthiest subjects. Subsequently, we identified non-overlapping groups of patients using ICD10 codes. We included 7 relevant groups of CVD: aortic stenosis (AS), atrial fibrillation (AF), cardiac sarcoidosis, chronic coronary artery disease (CAD), dilated cardiomyopathy (DCM), hypertrophic cardiomyopathy (HCM) and hypertension (without a history of other CVD, cardiovascular risk factors or other systemic diseases).

In addition, for the HCM group, we computed the LV mass (LVM) and LV ejection fraction (LVEF) and excluded any subject with LVM ≤2SD above the healthy population LVM mean or EF ≤45% [23]. For the DCM group, we computed the LV end-diastolic volume (LVEDV), end-systolic volume (LVESV) and LVEF and excluded any subject with an LVEF >45% or LVEDV ≤2SD above the healthy subjects’ EDV mean [24]. All volumetric metrics were computed using the automated method described in [10]. Finally, an experienced cardiologist reviewed the most unclear cases.

As pre-processing, all of the CMR digital imaging and communication in medicine (DICOM) images were converted into NIfTI format. The segmentations used for training and testing the PHiSeg network were performed by a cardiologist with 5 years’ CMR experience. To compute inter-observer variability, a subset of 30 subjects was segmented by two cardiologists with 5 years’ CMR experience and an experienced T_1_ mapping image analysis reader supervised by an external blinded experienced cardiologist. The LV endocardial and epicardial borders and the right ventricle (RV) endocardial border were traced using the ITK-SNAP interactive image visualisation and segmentation tool [25]. For training and testing the QC network, manual quality ratings were performed by a cardiologist with 5 years’ CMR experience using a user interface similar to Fig. 4 (i.e. a panel with two images: T_1_ map overlaid with its automated segmentation and the uncertainty map). To compute inter-observer variability, a subset of 300 subjects was labelled by two cardiologists with 5 years’ CMR experience and an experienced T_1_ mapping image analysis reader supervised by a external blinded experienced cardiologist.

### Automated image analysis

**Fig. 1:**
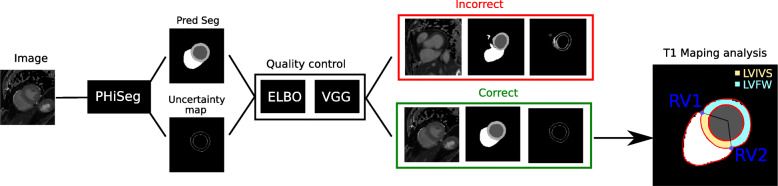
Overview of the proposed framework for automatic T_1_ map analysis. A composition of (1) the PHiSeg network for segmenting native T_1_ maps, (2) a QC for detecting inaccurate segmentation, and (3) T_1_ analysis. PHiSeg: Probabilistic Hierarchical Segmentation

The proposed workflow for automated T_1_ map analysis is summarised in Fig. 1 and described in detail in the following subsections.

#### Deep neural network with bayesian inference for segmentation

In this work, we used a Probabilistic Hierarchical Segmentation (PHiSeg) network [1], a recently proposed deep learning network with Bayesian inference for segmentation of the LV blood pool, LV myocardium and RV blood pool from T_1_ mapping images (Fig. 1). The PHiSeg network belongs to the group of probabilistic graphical models and employs convolutions to learn task-specific representations of the input data and predicts a pixel-wise segmentation from an input image based on this representation. In addition, an uncertainty map is generated which quantifies the pixel-wise uncertainty of the segmentation.

The PHiSeg network [1] models the probability distribution *p*(**S**|**X**) of plausible segmentations **S** for a given input image **X** at multiple scales from fine to coarse. Performing inference in this model using a conditional variational autoencoder approach results in a network architecture resembling the commonly used U-Net. However, in contrast to a U-Net, this network allows modelling of the joint probability of all pixels in the segmentation map. Specifically, after training it allows sampling of multiple plausible segmentation hypotheses for an input image. In this manner, in addition to producing a per-pixel prediction of the label class, it also allows estimation of the uncertainty corresponding to each pixel. The network architecture features a number of convolutional layers, each using a 3x3 kernel and a rectified linear unit (ReLU) activation function. After every three convolutions, the feature map is downsampled by a factor of 2 to learn more global scale features. After performing probabilistic inference at each level, the learned features are upsampled and fused to produce a predicted segmentation mask and a uncertainty map at the original image resolution. In line with the variational autoencoder literature [26] training the model amounts to finding the network parameters which maximise a lower bound on the log likelihood log*p*(**S**|**X**) given the training data. This quantity is typically referred to as the evidence lower bound (ELBO). The assumption is that with sufficient network complexity the ELBO will be a close-enough approximation of the distribution log*p*(**S**|**X**) to act as a surrogate for it. Specifically, after training we may evaluate the training objective (i.e. the ELBO) for a given image/segmentation pair to obtain its log likelihood. This may be used to identify segmentations which are very unlikely given an input image. A detailed description of the method, as well as the network architecture can be found in [1].

#### Network training and testing

For training of the PHiSeg network, all images were cropped using the manual segmentations to the same size of 192 ×192 and intensity normalised to the range [0,1]. Data augmentation was performed on-the-fly using random translations (±30 pixels), rotations (±15^∘^), flips (50% probability), scalings (up to 20%) and intensity transformations by gamma correction (*γ*∈ [0, 1.5]) to each mini-batch of images before feeding them to the network. The probability of augmentation for each of the parameters was 50%. Applying these transformations essentially produces a further expanded dataset which contains increased image variation preventing the network from fixating on features in specific regions of its receptive field. Augmentation is the only technique we use to prevent over-fitting, other techniques like dropout were found not to improve performance and so omitting them contributed to a simpler network architecture. Each mini-batch consisted of 20 native T_1_ map. To optimise the loss function we used the Adam optimiser, with the momentum set to 0.9 and the learning rate to 10^−3^. The models were trained for 50,000 iterations on a GeForce GTX TITAN GPU (NVIDIA Corporation, Santa Clara, California, USA) and the model with highest average Dice score (on the validation set) over all classes was selected. The total number of model parameters to learn was 18,709,372.

From the selected study population, we selected the following subjects for training/testing of the PHiSeg network:

- **Training database**: 800 subjects, consisting of both healthy subjects as well as subjects with a wide variety of CVDs. During training we used an 80/20 training/validation split to monitor the performance of the network.
- **Test database**: 100 subjects (50 healthy subjects and 50 chronic cardiomyopathy subjects).

#### Generating uncertainty maps

During test time, we used the PHiSeg network to sample *T* different segmentation output samples for a single given input (we used *T*=100). From these multiple segmentations the final predicted segmentation was calculated as the average softmax probability over all of the segmentation samples. We also decomposed the uncertainty map into a separate map for each segmentation class (i.e. background, LV blood pool, LV myocardium and RV blood pool) by computing the cross entropy between the mean segmentation mask and the different segmentation output samples for each of the different classes.

#### Quality control

Our QC process comprises two steps, both based upon different aspects of the uncertainty information provided by the PHiSeg network. To train these QC steps, we manually labelled the PHiSeg-obtained segmentations as correct or incorrect in a cohort of 800 subjects (consisting of both healthy subjects as well as subjects with a wide variety of CVDs).

First, we used the ELBO output [1,26] of the trained PHiSeg network to reject uncertain segmentations. The ELBO quantifies how likely it is that the segmentation is correct, and can detect very unlikely cases. However, it cannot detect cases with minor localised errors (e.g. inaccurate regional border). We used the manual QC labellings to determine a threshold and any ELBO value above this threshold resulted in the segmentation being rejected.

To increase the sensitivity of our QC process with respect to localised errors, we used a second QC step which is defined as an image classification problem, where each image/segmentation pair is classified as accurate or inaccurate. The outputs of the PHiSeg network (i.e. the image, segmentation and myocardial uncertainty map) were used as input to a deep learning image classifier. For the image classifier, we used a VGG-16 CNN network [27], which consists of a stack of convolutional layers followed by three fully-connected layers for classification. Each convolutional layer uses a 3x3 kernel and is followed by batch normalisation and ReLU. Details of the VGG-16 network can be found in [27]. Data augmentation was performed on-the-fly using random translations (±30 pixels), rotations (±15^∘^), flips (50% probability), scalings (up to 20%) and intensity transformations by gamma correction (*γ*∈ [0, 1.5]) to each mini-batch of images before feeding them to the network. The probability of augmentation for each of the parameters was 50%. To take into account the class imbalance between correct/incorrect samples we used a weighted binary cross entropy loss function. The VGG model’s performance was evaluated using a receiver operating characteristic curve (ROC) and the optimal classifier was selected using the Youden index. The model was trained for 50,000 iterations on a GeForce GTX TITAN (NVIDIA Corporation) and the model with highest accuracy (on the validation set) was selected. The total number of model parameters to learn was 11,177,025.

The following subjects were used for training/testing of the QC steps:

- **Training database**: 800 subjects, consisting of both healthy subjects as well as subjects with a wide variety of CVDs. During training we used an 80/20 training/validation split to monitor the performance of the network. The numbers of correct/incorrect segmentations were 132/668.
- **Test database**: 700 subjects (500 healthy subjects and 200 chronic cardiomyopathy subjects). The numbers of correct/incorrect segmentations were 116/584.

Data are rejected if either of these two QC steps fails. The combination of these two steps ensures that T_1_ maps acquired on different planes or with inaccurate segmentations are identified and rejected for further analysis.

#### T_1_ map analysis

Myocardial T_1_ values were measured from the mid-ventricular SAX slice for the whole myocardium, as well as for the interventricular septum and free-wall segments separately. From the predicted segmentation, the RV-LV intersection points were automatically detected from the LV/RV segmentation masks (RV1 and RV2 in Fig. 1) using the hit-or-miss transform, which is a morphological operation that detects a given configuration (or pattern) in an image. In our case, we aimed to detect the intersection between background, LV myocardium and RV labels. These RV-LV intersections were used to divide the LV myocardium mask into a LV interventricular septum (LVIVS) mask and a LV free-wall (LVFW) mask.

#### Myocardial t_1_ blood correction

For myocardial T_1_ blood correction, we used the model proposed by Nickander *et al* [6], which used a linear correction between myocardial T_1_ and blood measurements as follows:

1 $$ \mathrm{T}_{1}^{\text{corrected}} = \mathrm{T}_{1}^{\text{uncorrected}} + \alpha \cdot(\mathrm{R}_{\text{mean}} - \mathrm{R}_{\text{patient}}),   $$

where T_1_^uncorrected^ is the native myocardial T_1_ value, R _mean_ is the mean R_1_ for the patient cohort, and *α* is calculated as the slope of the linear regression between myocardial T_1_ and blood T_1_ measurements.

**Fig. 2:**
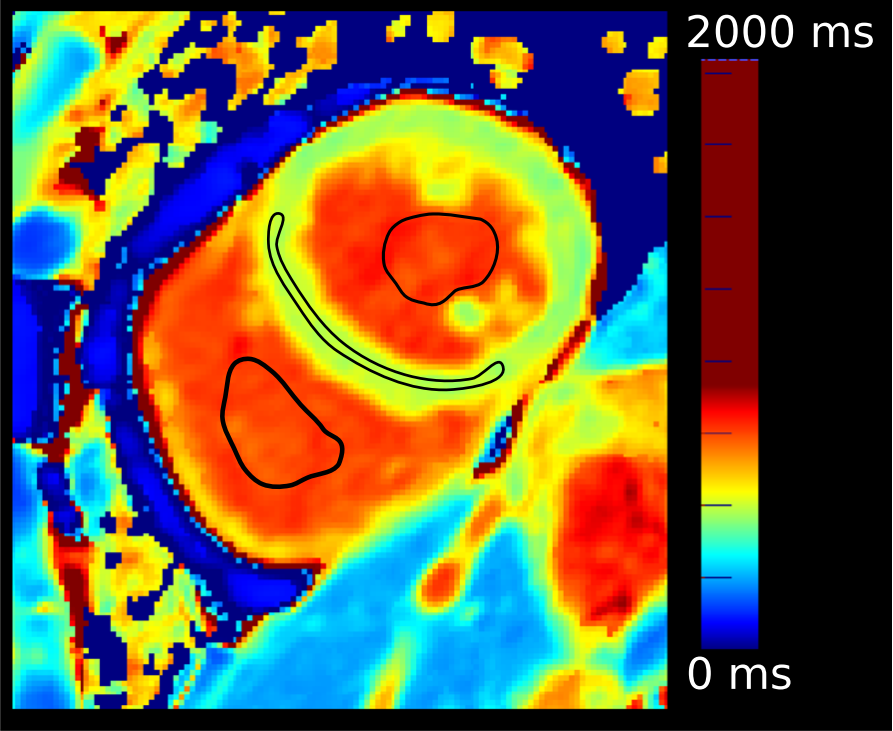
Short axis (Sax) T_1_ map with region-of-interest (ROIs). The figure shows the ROI drawn for native myocardial T_1_ values of the left ventricular (LV) intraventricular septum (IVS), LV and right ventricular (RV) blood pool T_1_ values

The blood T_1_ value was computed from RV and LV blood pool regions of interest (ROI) in the mid-ventricular SAX T_1_ map. To generate the LV and RV ROIs we eroded the LV/RV blood pool segmentations to generate a mask that has 1/3 of the area of the original mask (see Fig. 2). To ensure that no papillary muscles or trabeculae were included, we rejected any pixel whose T_1_ value was less than 1.5 times the interquartile range below the first quartile of the blood pool values. The blood T_1_ value was calculated as the mean of the LV and RV values calculated in this way, and then converted to the T_1_ relaxation rate (R_1_=1/T_1_). A similar strategy was used to obtain the ROIs and compute the global and regional T_1_ values from the manual segmentations (see subsection “UK biobank dataset” section and Fig. 2).

### Reference values

In total, we analysed CMR scans of 11,882 subjects (62 ±22 yrs., 48% males) included in the UK Biobank cohort using our method. First, we derived reference T_1_ values from 4,148 healthy subjects, selected using stringent criteria to exclude any disease or risk factor that impacts the heart or vasculature (see exclusion criteria in Additional file 1: Table 1). Next, we derived T_1_ values from patients known to have one of 7 different CVDs. Outliers for the computed T_1_ values were defined a priori as values 3 interquartile ranges below the first or above the third quartile and they were removed from the analysis. In addition, in order to allow comparison of cardiac functional metrics across the different groups, we computed LVEDV index (LVEDVI) LVEF and LVM index (LVMI) from cine CMR data, using the automated method described in [10]. For the healthy, hypertension and obesity groups the 10-year Framingham Risk Score was computed [22].

#### Evaluation of the method

We evaluated the performance of our automated method as follows:

**Deep neural network with Bayesian inference**: To validate the PHiSeg network, a cohort of 50 healthy subjects and 50 chronic cardiomyopathy patients was selected and manually segmented. These subjects were not used for training the PHiSeg network. We used the Dice metric to measure the degree of overlap between the automated and manual segmentations. The Dice metric has values between 0 and 1, where 0 denotes no overlap and 1 denotes perfect agreement. Furthermore, Bland-Altman analysis and Pearson’s correlation were used to compare the obtained global LV native myocardial T_1_ values, and the T_1_ values in the LV interventricular septum (IVS) and LV free wall (FW), between the automated and manual segmentations.

We also quantitatively assessed the inter-observer variability between manual segmentations by three clinical experts. From the test database, a subset of 30 subjects (15 healthy subjects and 15 chronic cardiomyopathy) was randomly selected and each subject was manually segmented by three clinical expert observers (O1, O2, O3) independently. The Dice metric and the global and regional T_1_ values were evaluated between each pair of observers (O1 vs O2, O2 vs O3, O3 vs O1).

**Quality control**: To assess the accuracy of the QC process, we manually labelled the PHiSeg obtained segmentations as correct or incorrect in a cohort of 500 healthy subjects and 200 chronic cardiomyopathy patients, which are independent from the training cohort. We computed sensitivity (% of manually labelled as incorrect image/segmentation pairs that were correctly detected), specificity (% of manually labelled as correct image/segmentation pairs that were correctly identified), and balanced accuracy (averaged percentages of correct answers for correct/incorrect classes individually).

We also assessed the inter-observer variability between manual labelling by three clinical experts. From the test database, a subset of 300 subjects (225 healthy subjects and 75 chronic cardiomyopathy) was randomly selected and each subject was analysed by three clinical expert observers (O1, O2, O3) independently. The intraclass correlation coefficient (ICC) and the Cohen’s kappa coefficient *k* were computed to measure the inter-rater agreement between each pair of observers (O1 vs O2, O2 vs O3, O3 vs O1).

#### Statistical analysis

Statistical analysis was performed using Statsmodels, a Python library for statistical and econometric analysis [28]. Normality of distributions was tested with the Kolmogorov-Smirnov test. Categorical data are expressed as percentages, and continuous variables as mean ± standard deviation (SD) or median and interquartile range, as appropriate. Paired 2-tailed Student’s *t*-tests were used to assess paired data, and unpaired 2-tailed Student’s *t*-tests were used to assess unpaired samples. Comparison of more than three normally distributed variables was performed using analysis of variance (ANOVA, with Bonferroni’s post-hoc correction). For the Bland-Altman analysis, paired *t*-tests versus zero values were used to verify the significance of the biases, and paired *t*-tests were used to analyse the mean absolute errors of all parameters between healthy subjects and patients. Linear regression was performed to estimate the slope used for correction of myocardial T_1_ from blood T_1_. The relationships between corrected and uncorrected mean native myocardial T_1_ were investigated by computing the SD of the mean native myocardial T_1_, and evaluated for difference with a F-test. To investigate whether blood correction improved the discrimination between healthy subjects and patients with CVDs, we calculated the z-scores of the patients in each CVD group with respect to our healthy population for the uncorrected and corrected T_1_ maps and compared the average z-scores using a paired 2-tailed Student’s *t*-test. Associations between native T_1_ values, clinical demographics and LV function were explored by single and multivariate linear regressions. In all cases, p <0.05 denotes statistical significance. To compute reference values, healthy subjects were used as the study controls and unpaired t-tests were used for comparison.

## Results

### Deep neural network with bayesian inference

Table 1 reports the Dice scores between automated and manual segmentations evaluated on the cohort of 100 subjects for the proposed method and a comparative model based on the U-Net architecture [29]. We also present Dice scores between the manual segmentations by different observers. Overall, the Dice score between manual and automated segmentations for the proposed method was 0.84 for the LV myocardium, which is close to or even smaller than the human-human difference.

**Table 1:** Dice scores between automated segmentation and manual segmentation for native T_1_ maps for the PHiSeg and U-Net segmentation networks, as well between segmentations by different human observers. The first two columns show the difference between automated and manual segmentations on a test set of 100 subjects. The third to fifth columns show the inter-observer variability, which is evaluated on a randomly selected set of 30 subjects (15 healthy subjects and 15 chronic cardiomyopathy subjects), each being analysed by three different human observers (O1, O2, O3) independently. Dice values are reported as mean (SD)

	**PHiSeg Auto vs Manual**	**U-Net Auto vs Manual**	**O1 vs O2**	**O1 vs O3**	**O2 vs O3**
	(N=100)	(N=100)	(N=30)	(N=30)	(N=30)
LV Blood pool	0.95 (1.63)	0.89 (7.96)	0.94 (2.22)	0.91 (2.83)	0.95 (1.66)
LV Myocardium	0.84 (3.16)	0.78 (8.89)	0.77 (7.57)	0.77 (6.53)	0.83 (2.56)
RV blood pool	0.92 (4.14)	0.81 (15.96)	0.84 (13.86)	0.90 (5.47)	0.91 (6.55)

LV, left ventricular; RV, right ventricular

**Fig. 3:**
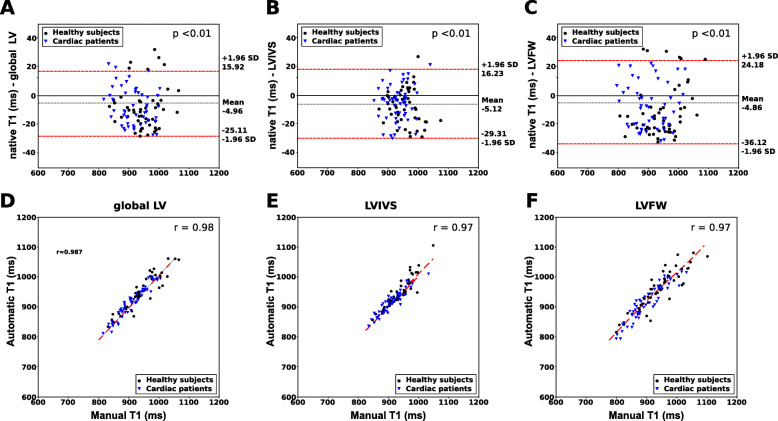
Bland-Altman and correlation plots of the automatic versus manual myocardium T_1_ values. **a**, **d** global LV native T_1_ values, (**b**, **e**) LVIVS native T_1_ values, (**c**, **f**) LV free wall (FW) native T_1_ values. In the Bland-Altman analysis, the grey dotted line represents the mean bias; the red dotted lines the limits of agreement. The p −values represent the difference in mean bias from zero using a paired *t*-test. In the correlation plots, the red dotted line represents linear regression line. *r* is the Pearson’s correlation coefficient

The Bland-Altman plot showed strong agreement between the pipeline and manual analysis, see Fig. 3. There was a small negative bias for all of the native T_1_ values (-5.04 ms, 5.89 ms and -4.97 ms for global LV, LVIVS and LVFW respectively). Table 2 shows the automatically and manually calculated T_1_ values within the three regions averaged over the test cohort. There was no significant difference in mean absolute error between manual and automated T_1_ values except for LVIVS T_1_ values for chronic cardiomyopathy patients. The automatically reconstructed T_1_ maps showed a strong correlation with the T_1_ values based on manual segmentations (r=0.97) (See Fig. 3). Finally, Table 3 reports the mean absolute difference between automated and manual T_1_ values and between T_1_ values by different expert observers. It shows that for the clinical measures, the computer-human difference is on a par with the human-human difference.

**Fig. 4:**
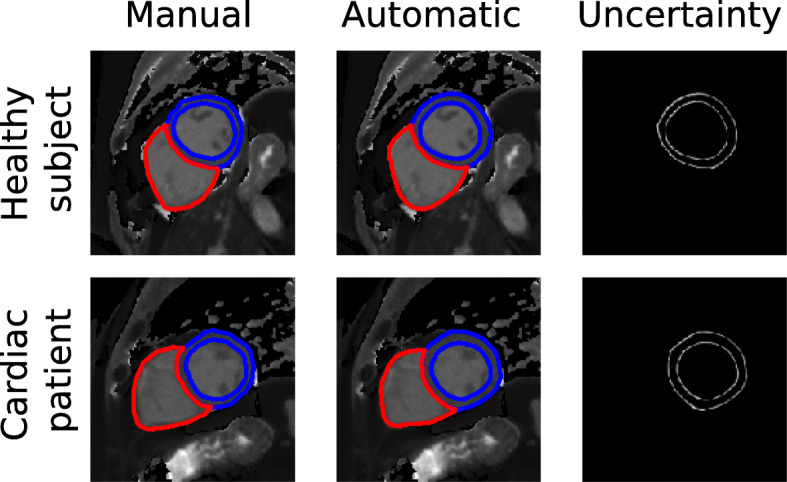
Illustration of the segmentation results for native T_1_ maps. The top row shows an example of a healthy subject and the bottom row of patient with cardiac disease. The cardiac chambers are represented by different colours

**Table 2:** Mean T_1_ values for the test cohort. T_1_ are reported as mean (SD). Asterisks indicate significant differences between T_1_ values estimated between manual and predicted segmentations using a paired *t*-test

	**Healthy**			**Chronic cardiomyopathy**		
	global LV	IVS	FW	Global LV	IVS	FW
Manual	945 (54)	952 (41)	941 (65)	911 (48)	930 (39)	902 (58)
Automatic	955 (66)	960 (58)	959 (72)	921 (51)	935 (37)*	913 (59)

**Table 3:** The difference in mean T_1_ between automated segmentation and manual segmentation, as well as between measurements by different human observers. The first two columns show the difference between automated and manual segmentations on a test set of 100 subjects for the proposed PHiSeg and U-Net networks. The third to fifth columns show the inter-observer variability, which is evaluated on a randomly selected set of 30 subjects, each being analysed by three different human observers (O1, O2, O3) independently. The mean and standard deviation (in parentheses) of the absolute difference and relative difference are reported

	**PHiSeg Auto vs Manual**	**U-Net Auto vs Manual**	**O1 vs O2**	**O1 vs O3**	**O2 vs O3**
	(N=100)	(N=100)	(N=30)	(N=30)	(N=30)
Global LV	12.40 (12.68)	75.75 (83.43)	17.03 (14.40)	20.53 (16.71)	19.18 (14.39)
LVIVS	13.11 (18.05)	81.33 (59.85)	19.23 (15.30)	17.88 (16.58)	21.12 (14.72)
LVFW	23.15 (12.85)	87.68 (164.32)	30.64 (15.02)	30.35 (18.32)	22.65 (19.03)

Figure 4 shows an example of a manual segmentation, the predicted segmentation and the uncertainty map for a healthy subject and for a chronic cardiomyopathy patient. Note that the manual and the automatic segmentations agree well. Additional file 2: Figure A2 presents an illustration of the PHiSeg latent space by showing the 100 segmentation samples for a sample subject. In general, the major differences in the different samples are in the boundaries between the different labels.

### Quality control

For the first QC step, the median and interquartile range (IQR) values for the ELBO were -502 and 215. A single threshold was selected based on the training data to maximise the accuracy of detecting wrong cases. In this case the selected threshold was -540. Table 4 shows the classification results of the different QC steps.

**Table 4:** QC classification sensitivity (SEN), specificity (SPE) and balanced accuracy (BACC) of the pipeline in detecting inaccurate or unusual output versus correct output with respect to manual assessment are shown. Q1 refers to the ELBO classifier, Q2 referes to the VGG network

	**BACC**	**Sensitivity**	**Specificity**
Q1	70.5	63.5	77.4
Q2	85.5	90.5	80.4
Q1 + Q2	93.1	95.3	90.9

From the test database, we manually categorised errors detected by the 2-step QC process as: (1) incorrect myocardium segmentation, (2) incorrect planning, (3) motion artefacts. The percentages of subjects for each of these categories were 19.3%, 18.1% and 62.6% respectively, showing that approximately 80% of cases were medically untreatable, i.e. the T_1_ maps were either affected by motion artefacts or acquired from an incorrect plane. The cases that could not be detected by either of the steps tended to be those that had minor errors in the segmentation of the myocardium.

The ICCs between observers (O1, O2, O3) were ICC _*O*1−*O*2_=0.82, ICC _*O*1−*O*3_=0.84 and ICC _*O*2−*O*3_=0.89. The Cohen’s kappa coefficients between observers (O1, O2, O3) were *k*_*O*1−*O*2_=0.83,*k*_*O*1−*O*3_=0.85 and *k*_*O*2−*O*3_=0.88. The Cohen’s kappa coefficients show a strong correlation, and the ICC indicates good reliability between the measures provided by the different observers.

**Fig. 5:**
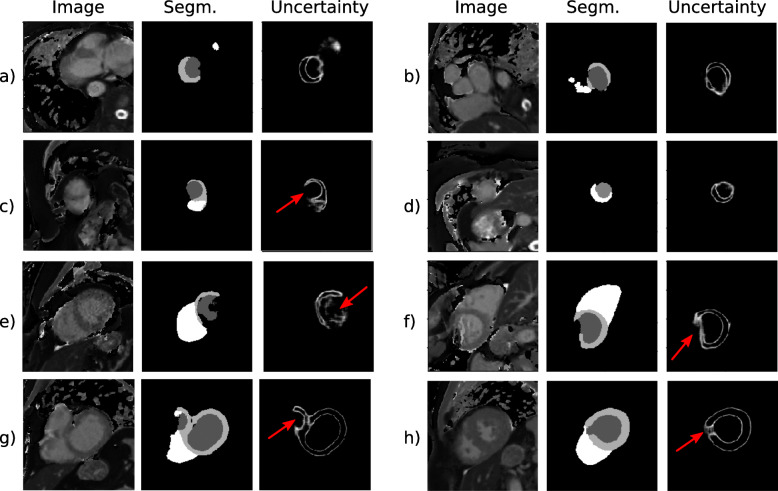
Segmentation and uncertainty map results for selected subjects who were rejected by the quality control QC process. The left column represents the T_1_ mapping images, the middle column the automated segmentation, and the right column the derived uncertainty map. Red arrows indicate the regions with high uncertainty

Figure 5 shows examples of uncertainty maps for a cohort of subjects that have been rejected by the QC steps. Note that the QC is able to identify inaccurate data with a wide range of underlying causes such as incorrect planning (i.e. images (a), (b), (c) and (d) in Fig. 5), motion artefacts (i.e. images (e) and (f) in Fig. 5) or segmentation failure (images (g) and (h) in Fig. 5).

### Uncertainty quantification

To understand the impact of uncertainty in the predicted T_1_ values, from the test cohort that contains 50 healthy subjects and 50 chronic cardiomyopathy patients, we computed the distribution of the global LV T_1_ values over the *T* predicted segmentations. Additional file 3: Figure 1 shows a graphical representations of the variability of these estimates. The solid lines indicate the mean T_1_ values from the test cohort and the shaded region represents one SD of uncertainty.

### Myocardial t_1_ blood correction

The constants from the linear regression model between the myocardial T_1_ and the blood measurements were used to correct native myocardial T_1_ values according to Equation 1. The global R^2^ was 0.29 (which is comparable to that found in [6]) and the mean squared error was 101. The linear regression had a slope of -0.36 and an intercept of 925.

The mean uncorrected myocardial T_1_ in the population cohort was 951 ± 49, and the mean corrected myocardial T_1_ in the population cohort was 921 ± 44, showing a statistically significant decrease of the SD (p <0.001).

#### Reference values

From the 11,882 subjects, 1865 (15.7%) subjects were rejected by our QC process due to inaccurate T_1_ segmentations. Subject characteristics of the remaining 10,017 subjects are summarised in Table 5. Compared to healthy subjects, all patient groups were in the same age range; patients with obesity and cardiac sarcoidosis had statistically higher BMI and body surface area (BSA); patients with DCM and chronic coronary artery disease had significantly lower LVEF (all p <0.05); patients with HCM and DCM had significantly higher iLVM (all p <0.05).

**Table 5:** Baseline characteristics of study subjects included in the analysis for reference values. All values are n (%) or mean ± SD. Abbreviations: AF (atrial fibrillation), AS (aortic stenosis), BMI (body mass index), BSA (body surface area), g (grams), CAD (coronary artery disease), DCM (dilated cardiomyopathy), HCM (hypertrophic cardiomyopathy), HR (heart rate), kg (kilograms), LVEDVI (LV end-diastolic volume index), LVEF (left ventricular ejection fraction), LVMI (LV mass index), m (metre). Disease names are as per abbreviations list. * denotes values significantly different from healthy subjects (all p <0.05)

	n	Age (years)	Male (n)	BMI (kg/m^2^)	BSA (m^2^)	HR (bpm)	iLVEDV(mL/m^2^)	LVEF (%)	iLVM (g/m^2^)	Framingham Risk Score (%)
Healthy	4148	61 (7)	2299 (55.42)	24 (3)	1.82 (0.2)	61 (10)	78 (13)	59 (5)	45 (8)	5.9 (3.2)
AS	30	68 (7)	15 (29.41)	28 (4)	1.97 (0.2)	73 (5)	83 (23)	59 (6)	56 (13)	
AF	527	68 (7)	293 (55.60)	27 (4)	1.87 (0.2)	62 (12)	83 (23)	58 (10)	50 (14)	
Cardiac Sarcoidosis	70	59 (7)	40 (57.14)	29 (7)*	1.97 (0.2)*	61 (5)	72 (18)	58 (4)	41 (11)	
Chronic CAD	1204	69 (6)	624 (51.83)	27 (4)	1.88 (0.2)	58 (11)	84 (25)	56 (10)*	49 (15)	
DCM	260	66 (6)	150 (57.69)	26 (3)	1.84 (0.2)	63 (14)	111 (42)*	44 (14)	59 (22)*	
HCM	50	64 (5)	30 (60.00)	23 (2)	1.79 (0.2)	60 (15)	88 (35)	62 (14)	79 (14)*	
Hypertension	3313	63 (7)	1722 (51.98)	27 (4)	1.88 (0.2)	64 (11)	80 (21)	59 (7)	50 (14)	11.9 (7.1)*
Obesity	415	60 (7)	213 (51.33)	33 (3)*	2.05 (0.2)*	63 (10)	75 (14)	58 (6)	46 (9)	6.9 (3.2)*

**Fig. 6:**
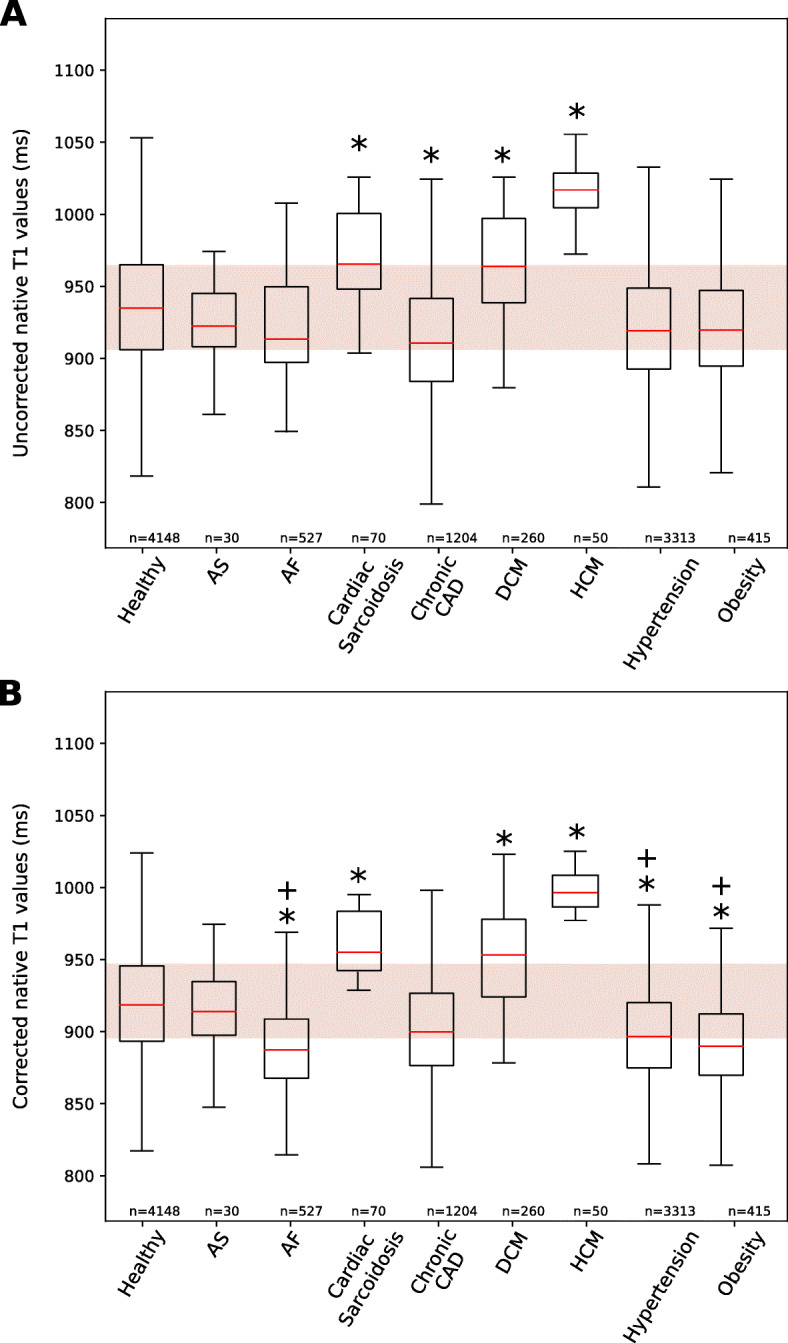
Box plot for uncorrected and corrected LVIVS T_1_ for 7 different cardiovascular conditions and obese/healthy subjects. Characteristic native LVIVS for (**a**) uncorrected and (**b**) corrected T_1_ values (1.5 T). Data presented as box and whisker plots with the median, upper and lower quartiles, min and max excluding outliers, and outliers that are more than 3/2 the upper and lower quartiles. Disease names are as per abbreviations list. * denotes values significantly different from healthy subjects and *†* denotes a significantly decrease of *z*-score from the uncorrected LVIVS T_1_ values using a paired *t*-test (all p <0.05)

Uncorrected and corrected myocardial LVIVS T_1_ values for the different CVD groups and in healthy subjects are shown in Fig. 6. Table 6 shows global and regional ((LV, LVIVS, LVFW)) native corrected and uncorrected T_1_ values. Compared to healthy subjects, patients with HCM, DCM and cardiac sarcoidosis had significantly higher native T_1_ values, while patients with AF, hypertension and obesity had significantly lower native T_1_ values. Gender did not affect myocardial T_1_ values significantly (2-way ANOVA, p = 0.01), and thus only overall T_1_ values are reported.

**Table 6:** Reference ranges for the native uncorrected and corrected ShMOLLI-T_1_ values. Reference ranges for the most common myocardial tissue conditions encountered in clinical practice. Abbreviations: LV IVS (LV interventricular septum), LVFW (LV free-wall). Disease names are as per abbreviations list. *denotes values significantly different from healthy subjects (all p <0.05)

		**Uncorrected ShMOLLI-T**_**1**_**ranges**		
	n	global LV T_1_	IVS T_1_	FW T_1_
Healthy	4148	932 (50)	937 (49)	930 (55)
Aortic stenosis	30	918 (29)	924 (35)	916 (34)
Atrial fibrillation	527	920 (46)*	925 (47)*	917 (51)*
Cardiac Sarcoidosis	70	980 (83)*	976 (70)*	984 (91)*
Chronic coronary artery disease	1204	908 (45)*	913 (46)*	906 (50)*
Dilated cardiomyopathy	260	975 (56)	972 (39)	973 (66)
Hypertrophic cardiomyopathy	50	999 (24)*	1015 (20)*	992 (26)*
Hypertension	3313	914 (46)*	921 (45)*	910 (50)*
Obesity	415	910 (43)*	918 (43)*	906 (48)*
		**Corrected ShMOLLI-T**_**1**_**ranges**		
	n	global LV T_1_	IVS T_1_	FW T_1_
Healthy	4148	930 (45)	934 (43)	928 (50)
Aortic stenosis	30	922 (23)	928 (28)	920 (30)
Atrial fibrillation	527	919 (42)*	925 (43)*	917 (48)*
Cardiac Sarcoidosis	70	986 (75)*	982 (62)*	990 (83)*
Chronic coronary artery disease	1204	908 (42)*	913 (43)*	906 (47)*
Dilated cardiomyopathy	260	976 (53)	973 (36)	973 (63)
Hypertrophic cardiomyopathy	50	992 (21)*	1009 (28)*	986 (23)*
Hypertension	3313	917 (42)*	925 (41)*	914 (47)*
Obesity	415	914 (40)*	922 (40)*	910 (45)*

## Discussion

In this work, we have proposed a fully automated pipeline with a novel quality control step to automatically quantify myocardial tissue from native T_1_ mapping, which allows extraction of reference values from large-scale databases. The method is fast and scalable, overcoming limitations associated with current clinical CMR image analysis workflows, which are manual, time-consuming and prone to subjective error. The method has potential to automate T_1_ mapping analyses from CMR in clinical practice and research. Using the proposed pipeline we present reference ranges for global and regional myocardial native T_1_ in healthy subjects from the UK Biobank dataset and show that blood correction improves discrimination between healthy subjects and patients with CVD.

### Automatic analysis with quality control

We validated our segmentation network by comparing between automated and manual analysis in a cohort of healthy and diseased subjects. Results show a strong agreement for both segmentations (see Table 1) as well as estimated T_1_ values (Fig. 3).

Residual biases on the Bland-Altman plot are within the range of inter- and intra-observer variabilities reported in Tables 1 and 2 and in [30]. Also, residual biases (ranging between -4.97ms and -5.04ms) are consistent between healthy and CVD patients and are unlikely to have significant clinical impact. The Dice scores we obtained are comparable to previous works [11].

QC techniques are essential to be able to translate deep learning algorithms into a clinical setting. However, many works proposed for the analysis of CMR data have not taken this need into account making it impossible to deploy them for the processing of large-scale databases. In our framework, we employed a novel approach that used the uncertainty information produced by a CNN with Bayesian inference to identify incorrect segmentations, which can be rejected or flagged for revision by an expert cardiologist. We show that this QC process yields over 95% sensitivity in detecting errors.

### Reference values:

We used our framework to analyse native T_1_ maps in an unprecedentedly large cohort of healthy subjects and patients with CVD. Using this cohort we were able to provide reference values for normal myocardium in different CVD groups. T_1_ values differ between different scanners, vendors and protocols. Our values can therefore not directly compare to other publications, but they are in agreement with results obtained in smaller cohorts of manual assessment [8, 31–34]. One limitation of the presented T_1_ reference values for the healthy control group is the mean age of 61 years old, which is slightly higher than the mean age of other cohorts. We further used our cohort to interrogate T_1_ values in patients with 7 different CVDs. We show that for CVDs in which diffuse myocardial disease is prominent (cardiac sarcoidosis, HCM and DCM) we find significantly higher T_1_ values. For the other CVDs, we show that T_1_ values did not significantly change from healthy data. It is likely that the extent of myocardial damage in these groups is less high, explaining the lower T_1_ values. There is large variability seen in native T_1_ values, which is known to be caused by several factors, including intracardiac blood flow and hematocrite levels. We investigated a previously proposed method for correction of T_1_ values based on blood pool T_1_ dynamics [6]. This method has the benefit of working using image data alone, but has not previously been tested in a large cohort of patients. We demonstrate that indeed, discrimination between health and disease improves using blood pool correction. Whether this technique is better than using hematocrite-correction or other methods should be investigated in further studies. Investigating myocardial disease processes should include additional measures to native T_1_, such as extracellular volume or T_2_ imaging. However, the data presented in our study remain valuable. The UK Biobank cohort will contain highly detailed imaging and non-imaging data and follow-up in nearly half a million subjects. On this large scale, new relationships between T_1_ and population characteristics can yield important insights into development and progression of CVDs.

### Limitations and future directions:

The original PHiSeg paper aimed to capture uncertainty caused by different annotators’ segmentation styles as well as to capture inherent uncertainties due to factors such as poor contrast or other restrictions imposed by the image acquisition. In this work we only take advantage of the second contribution and this might result in a slightly lower performance. However, compared to the state-of-the-art U-Net network we showed that the proposed method has significantly higher Dice values and lower absolute errors for T_1_ values.

A limitation of our work is that the PHiSeg network was trained on a single dataset, the UK Biobank dataset, which is relatively homogeneous and only contains native T_1_ maps acquired using ShMOLLI on a 1.5T Siemens scanner. To obtain similar performance in other databases it would be necessary to retrain the networks using a small amount of data. However, the proposed segmentation model and QC steps will remain applicable.

Another limitation of this study is the lack of availability of paired LGE and native T_1_-mapping data to assess the correlation between these two measurements, and in which cases T_1_-mapping could provide a better insight into cardiac pathologies. Based on previous studies, it is known that T_1_-mapping may enable detection of early pathological processes, and serve as a tool for early diagnosis or screening, or differentiation of cardiomyopathies from normal phenotypes. The provided reference ranges could help to identity subjects at risk at an early stage.

The T_1_ values presented in this study were derived using a single T_1_-mapping technique. It is important to take into account that even within the same T_1_-mapping technique, different versions of sequences can lead to small differences in T_1_-estimations. Therefore, it might not be possible to directly translate the T_1_ values derived in this study to other T_1_-mapping techniques. In future work we aim to extend the automatic pipeline to be able to accurately segment T_1_-mapping data from different sequences/vendors, to make the proposed framework generalisable.

## Conclusions

We presented and validated a pipeline for automated quantification of myocardial tissue from native T_1_-mapping. The proposed method uses the uncertainty of a deep learning segmentation network in a novel QC process to detect inaccurate segmentations. We used the proposed framework to obtain reference values from the largest cohort of subjects to date, which include data from healthy subjects and from patients with the most common myocardial tissue conditions.

## Supplementary information

**Additional file 1** Table 1.

**Additional file 2** Figure A2.

**Additional file 3** Figure 1.

## Data Availability

The imaging data and manual annotations were provided by the UK Biobank Resource under Application Number 17806. Researchers can apply to use the UK Biobank data resource for health-related research in the public interest [35].

## Abbreviations

AF: Atrial fibrillation
ANOVA: Analysis of variance
AS: Aortic stenosis
BMI: Body mass index
bmp: Beats per minute
BSA: Body surface area
CAD: Coronary artery disease
CMR: Cardiovascular magnetic resonance
CNN: Convolutional neural networks
cVAE: Conditional variational autoencoder
CVD: Cardiovascular disease
DCM: Dilated cardiomyopathy
ELBO: Evidence lower bound
HCM: Hypertrophic cardiomyopathy
LVEDVI: Left ventricular end-diastolic volume index
iLVM: LV mass index
LGE: Late gadolinium enhancement
LVEF: LV ejection fraction
QC: Quality control
PHiSeg: Probabilistic Hierarchical Segmentation
ReLU: Rectified linear unit
ROI: Regions of interest
RV: Right ventricle/right ventricular
SAX: Short axis
SD: Standard deviation
DICOM: Digital imaging and communication in medicine
FW: Free wall
IVS: Intraventricular septum
LV: Left ventricle/left ventricular
LVEDV: Left ventricular end-diastolic volume
LVEF: Left ventricular ejection fraction
LVESV: Left ventricular end-systolic volume
LVESVI: Left ventricular end-systolic volume index
ROC: Receiver operating characteristic
ShMOLLI: Shortened modified Look-Locker inversion recovery
