# Supplementary material for: Automated quantification of myocardial tissue characteristics from native T1 mapping using neural networks with uncertainty-based quality-control
Source: J Cardiovasc Magn Reson. 2020 Aug 20;22:60. doi: 10.1186/s12968-020-00650-y (PMC7439533; doi:10.1186/s12968-020-00650-y)
Supplement: Supplementary file 1 — Additional file 1 Table 1. [file 12968_2020_650_MOESM1_ESM.pdf]

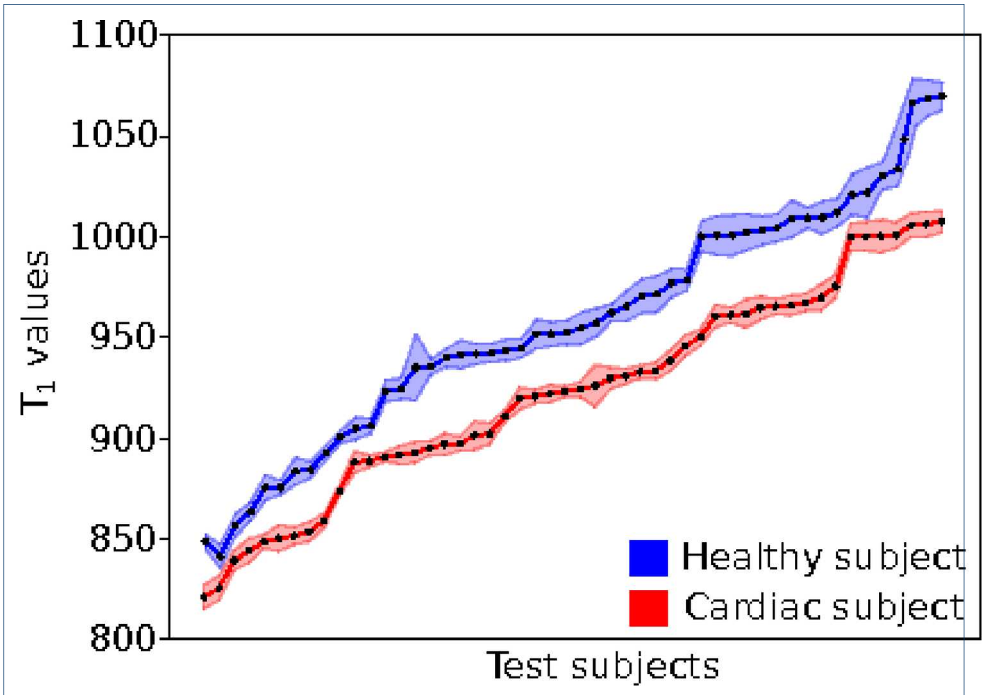

Fig. A.1: Uncertainty plot for predicted global LV T_1. Data presented in ascending order of predicted T_1 showing mean T_1 value and SD of uncertainty for each subject. Colours represent the different groups in the test cohort

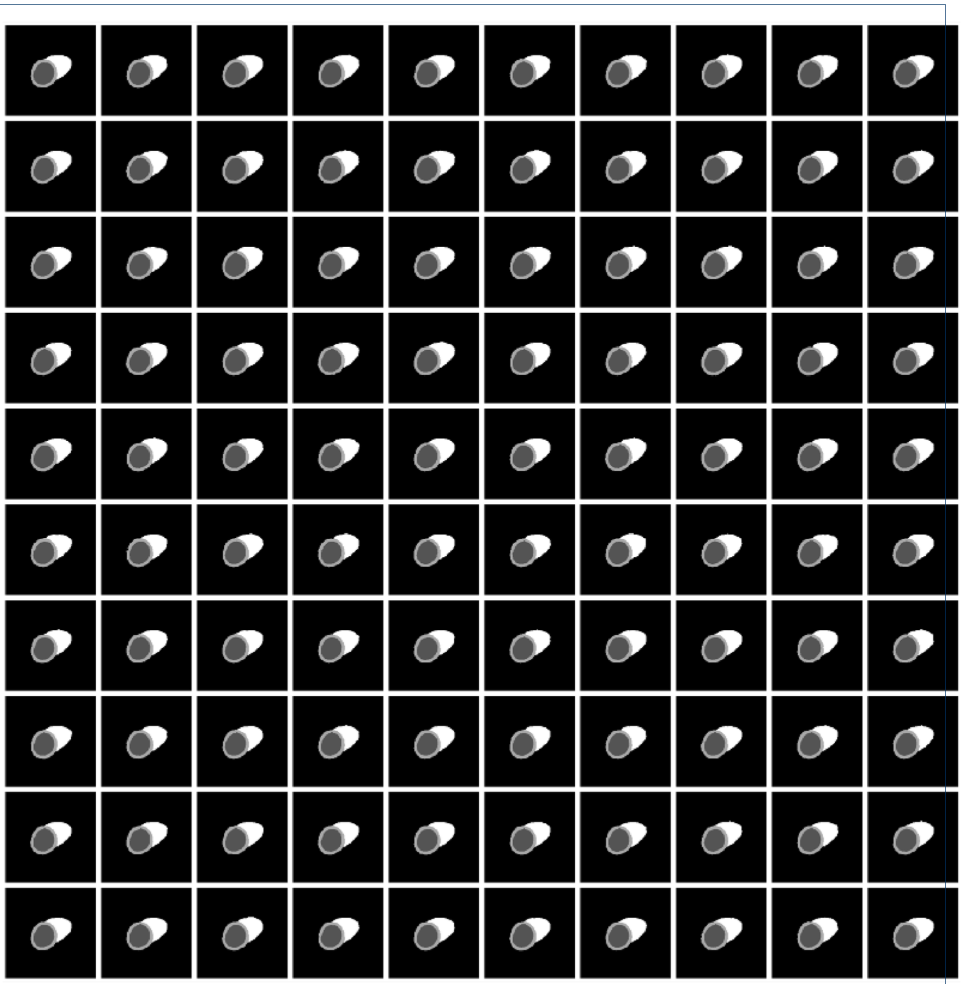

Fig. A.2: Illustration of the latent space showing an example of 100 samples generated for a subject.

Table A.1: Exclusion criteria for UK Biobank

**Age and body mass:** Age >74
**Medical conditions:** Adrenocortical insufficiency/Addison's disease, Alcoholic liver disease/alcoholic cirrhosis, Anaemia, Angina, Ankylosing spondylitis, Anorexia/bulimia/other eating disorder, Antiphospholipid syndrome, Aortic aneurysm, Aortic regurgitation/incompetence, Aortic stenosis, Aplastic anaemia, Asthma, Atrial fibrillation, Atrial flutter, Bronchiectasis, Cardiomyopathy, Chronic obstructive airways disease/COPD, Clotting disorder/excessive bleeding, Connective tissue disorder, Crohns disease, Diabetes, Diabetes insipidus, Diabetic eye disease, Diabetic neuropathy/ulcers, Doctor diagnosed bronchiectasis, Emphysema, Emphysema/chronic bronchitis, Essential hypertension, Fibrosing alveolitis/unspecified alveolitis, Gestational diabetes, Gestational diabetes only, Gestational hypertension/pre-eclampsia, Giant cell/temporal arteritis, Glomerulnephritis, Grave's disease, Haemochromatosis, Haemophilia, Heart arrhythmia, Heart attack/myocardial infarction, Heart failure/pulmonary edema, Heart valve problem/heart murmur, Heart/cardiac problem, Hereditary/genetic haematological disorder, High cholesterol, Hyperaldosteronism/Conn's syndrome, Hyperprolactinaemia, Hypertension, Hyperthyroidism/thyrotoxicosis, Hypertrophic cardiomyopathy (HCM / HOCM), Hypopituitarism, Hypothyroidism / myxoedema, Iga nephropathy, Inflammatory bowel disease, Interstitial lung disease, Iron deficiency anaemia, Irregular heart beat, Kidney nephropathy, Leg claudication/intermittent claudication, Liver failure/cirrhosis, Low platelets/platelet disorder, Lymphoedema, Microscopic polyarteritis, Miscarriage, Mitral regurgitation/incompetence, Mitral valve disease, Mitral valve prolapse, Monoclonal gammopathy/not myeloma, Myeloproliferative disorder, Myocarditis, Myositis/myopathy, Nephritis, Neutropenia/lymphopenia, Other respiratory problems, Pericardial effusion, Pericardial problem, Pericarditis, Peripheral vascular disease, Pernicious anaemia, Pleural effusion, Pleural plaques (not known asbestosis), Polycythaemia vera, Polymyalgia rheumatica, Polymyositis, Pulmonary embolism +/- DVT, Renal failure not requiring dialysis, Renal/kidney failure, Respiratory failure, Retinal artery/vein occlusion, Rheumatic fever, Sarcoidosis, Sick sinus syndrome, Sickle cell disease, Sjogren's syndrome/sicca syndrome, Sleep apnoea, Stroke, Surgery/amputation of leg above the knee, Surgery/amputation of leg below the knee, Surgery/amputation of toe, SVT/supraventricular tachycardia, Systemic lupus erythematosis/SLE, Transient ischaemic attack (TIA), Type 1 diabetes, Type 2 diabetes, Ulcerative colitis, Vasculitis, Wegners granulmatosis, Wolff parkinson white/WPW syndrome
**Medications:** Blood pressure medication, Cholesterol lowering medication, Hormone replacement therapy, Insulin
**Symptoms:** Chest pain due to walking ceases when standing still, Chest pain when walking uphill or hurrying, Shortness of breath walking on level ground, Unable to walk up hills or to hurry
**Smoking history:** Current smoker, Ex-smoker
**Ethnicity:** African, Any other Asian background, Any other Black background, Any other mixed background, Asian or Asian British, Bangladeshi, Black or Black British, Caribbean, Chinese, Do not know, Indian, Other ethnic group, Pakistani, Prefer not to answer, White and Asian, White and Black African, White and Black Caribbean
