# Supplementary figures and images for: Automated quantification of myocardial tissue characteristics from native T1 mapping using neural networks with uncertainty-based quality-control
Source: J Cardiovasc Magn Reson. 2020 Aug 20;22:60. doi: 10.1186/s12968-020-00650-y (PMC7439533; doi:10.1186/s12968-020-00650-y)

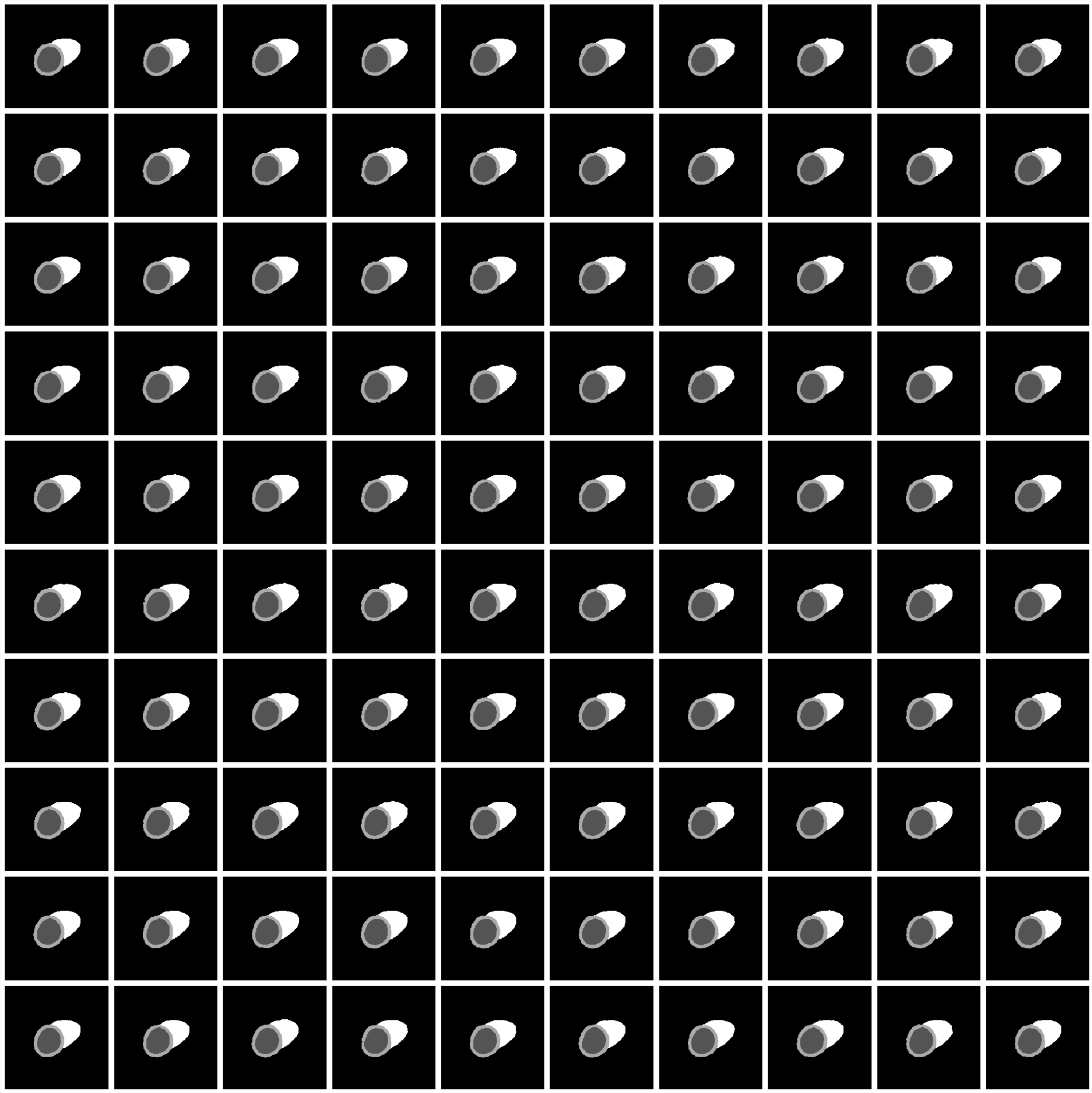

Supplement: Supplementary file 2 — Additional file 2 Figure A2. [file 12968_2020_650_MOESM2_ESM.pdf]

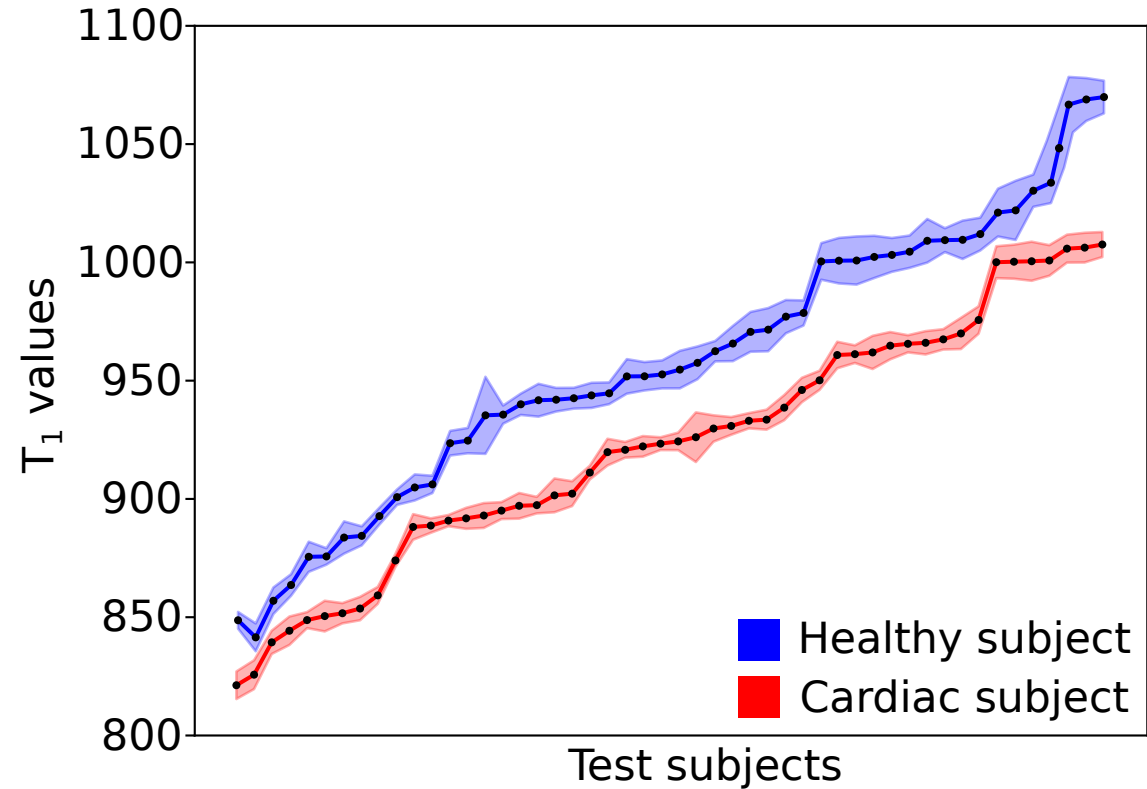

Supplement: Supplementary file 3 — Additional file 3 Figure 1. [file 12968_2020_650_MOESM3_ESM.pdf]
